# Circulating Fibroblast Growth Factor 21 is Associated with Subsequent Renal Injury Events in Patients Undergoing Coronary Angiography

**DOI:** 10.1038/s41598-018-30744-8

**Published:** 2018-08-20

**Authors:** Cheng-Hsueh Wu, Ruey-Hsing Chou, Chin-Sung Kuo, Po-Hsun Huang, Chun-Chin Chang, Hsin-Bang Leu, Chin-Chou Huang, Jaw-Wen Chen, Shing-Jong Lin

**Affiliations:** 1 Division of Cardiology, Department of Medicine, Taipei Veterans General Hospital, Taipei, Taiwan; 2 Division of Endocrinology and Metabolism, Department of Medicine, Taipei Veterans General Hospital, Taipei, Taiwan; 3 Department of Critical Care Medicine, Taipei Veterans General Hospital, Taipei, Taiwan; 4 Healthcare and Management Center, Taipei Veterans General Hospital, Taipei, Taiwan; 5 Department of Medical Education, Taipei Veterans General Hospital, Taipei, Taiwan; 6 Department of Medical Research, Taipei Veterans General Hospital, Taipei, Taiwan; 7 Cardiovascular Research Center, Taipei Veterans General Hospital, Taipei, Taiwan; 8 Institute of Clinical Medicine, National Yang-Ming University, Taipei, Taiwan; 9 Institute of Pharmacology, National Yang-Ming University, Taipei, Taiwan; 10 School of Medicine, National Yang-Ming University, Taipei, Taiwan

## Abstract

Fibroblast growth factor 21 (FGF21) is a regulator of glucose homeostasis, and is suggested to have protective effect on diabetic nephropathy. Its impact on non-diabetic kidney disease is unclear. To investigate the impact of FGF21 on contrast-induced nephropathy (CIN), 531 subjects underwent elective coronary angiography (CAG) were enrolled. Baseline creatinine and FGF21 were obtained before CAG. Patients were grouped into tertiles according to their FGF21 concentration. Creatinine was obtained 48 hours after CAG, and every 6 months in the follow-up period. Renal function decline was defined as >30% reduction of eGFR from baseline. All subjects were followed up till December 2016, or till the occurrence of major adverse cardiovascular events (MACE). Patients with higher FGF21 concentration were older, had higher incidence of hypertension, diabetes, chronic kidney disease, and heart failure. Thirty-four cases of CIN and 111 cases of renal function decline were identified during mean follow-up of 2.3 ± 1.3 years. Circulating FGF21 level was independently associated with CIN (aOR: 4.66, 95% CI: 1.29–16.86, *p* = 0.019) and renal function decline (aHR: 7.98, 95% CI: 4.07–15.66, *p* < 0.001) whether diabetes was present or not. In conclusion, circulating FGF21 level is independently associated with the incidence of CIN and subsequent kidney injury in patients undergoing CAG.

## Introduction

Contrast-induced nephropathy (CIN), an acute kidney injury occurring 2–7 days after the administration of contrast medium, is a common complication of cardiac catheterization. The incidence of CIN ranges widely, from 7% to 25%, depending on the presence of risk factors^1^ and baseline renal function. CIN was traditionally regarded as a benign and reversible disease, but increasing evidence suggests that it is associated with worsening short-term and long-term outcomes^2^. Moreover, CIN is related to prolonged hospitalization and increased medical costs and in-hospital mortality^3^. Epidemiologic data revealed that about 1% of CIN cases require in-hospital dialysis, with overall mortality rates of 7–31%^4^. Persistent renal damage occurs in about 20% of CIN cases, and persistent renal function impairment has been associated with a 5-fold increase in the risk of death at the 5-year follow-up^2^. Early identification of high-risk populations and prevention of CIN are thus important. However, except for adequate hydration and the administration of high-dose statins, no effective pharmacologic therapy is currently available for the prevention of CIN^5^.

Fibroblast growth factor 21 (FGF21) is a liver-secreted protein that acts as an endocrine factor in the circulation^6^. The physiologic function of FGF21 is to promote glucose uptake and fatty acid oxidation in adipocytes^7^. Emerging evidence suggests that FGF21 is a promising therapeutic target for diabetes and various metabolic disorders. Exogenous FGF21 treatment had been shown to suppress renal lipid accumulation and prevent diabetic nephropathy in animal studies^8^. However, the association of circulating FGF21 level with acute kidney injury and subsequent renal function decline has not been evaluated sufficiently in previous research. Therefore, we hypothesized that FGF21 would affect CIN and subsequent renal function deterioration in patients with stable angina who had been exposed to contrast media. In this single-center observational study, we measured circulating FGF21 concentrations and followed renal function in patients undergoing coronary angiography (CAG) and/or percutaneous coronary intervention (PCI) to investigate the role of FGF21 in the occurrence of CIN and chronic renal function decline.

## Results

### Baseline Characteristics

Of 588 subjects screened, 39 patients with end-stage renal disease (ESRD), 17 patients took fenofibrate, and 1 patient without detectable FGF21 value were excluded. The remaining 531 subjects, who underwent elective CAG and/or PCI, were enrolled in the study. The mean age of the study population was 68.6 ± 12.9 years, and 66.9% of the patients were male. Table 1 summarizes the clinical and demographic characteristics of patients according to FGF21 concentration. Patients with higher serum FGF21 concentrations were older and had higher incidences of hypertension, diabetes, chronic kidney disease (CKD), heart failure, and multiple vessel disease. Subjects in the tertile of highest FGF21 concentration were found to have the highest fasting glucose level, proteinuria; and the lowest hemoglobin level, estimated glomerular filtration rate (eGFR), and left ventricular ejection fraction (LVEF).

**Table 1:** Baseline Characteristics of the Study Cohort by Tertiles of Serum Fibroblast Growth Factor 21 Concentration.

Characteristic	Tertile 1 FGF21 < 113.7	Tertile 2 FGF21: 113.7–227.3	Tertile 3 FGF21 ≥ 227.3	*P*
Age (years)	65.3 ± 14.0	70.0 ± 12.3	70.5 ± 11.9	<0.001
Male, n (%)	135 (76.3)	117 (66.1)	103 (58.2)	0.001
Smoking, n (%)	62 (35.0)	56 (31.6)	63 (35.6)	0.697
BMI (kg/m^2^)	25.2 ± 4.1	26.0 ± 4.2	25.7 ± 4.4	0.209
Medical history, *n* (%)				
Hypertension	98 (55.4)	121 (68.4)	133 (75.1)	<0.001
Diabetes	39 (22.0)	65 (36.7)	80 (45.2)	<0.001
Chronic kidney disease	15 (8.5)	52 (29.4)	77 (43.5)	<0.001
Heart failure	7 (4.0)	14 (7.9)	27 (15.3)	0.001
Previous MI	9 (5.1)	11 (6.2)	11 (6.2)	0.872
Medications, *n* (%)				
Antiplatelet agents	91 (51.4)	105 (59.3)	91 (51.4)	0.226
ACEIs/ARBs	26 (14.7)	43 (24.3)	52 (29.4)	0.004
Diuretics	15 (8.5)	11 (6.2)	27 (15.3)	0.013
OHAs	17 (9.7)	35 (19.8)	27 (15.3)	0.028
Insulin	4 (2.3)	15 (8.5)	17 (9.7)	0.013
Statins	42 (23.7)	44 (24.9)	42 (23.7)	0.960
NAC prevention	3 (1.7)	6 (3.4)	27 (15.3)	<0.001
Laboratory data				
WBCs (K/cumm)	7.0 ± 1.9	7.4 ± 8.1	7.4 ± 2.5	0.637
Hemoglobin (g/dL)	13.3 ± 1.5	12.6 ± 1.7	12.2 ± 2.0	<0.001
Fasting glucose (mg/dL)	105.6 ± 28.9	119.5 ± 87.2	125.3 ± 48.1	0.011
HbA1c (%)	6.8 ± 1.5	6.8 ± 1.3	7.2 ± 1.6	0.117
Proteinuria (mg/dL)	5.9 ± 34.0	14.1 ± 45.2	30.5 ± 88.1	0.001
eGFR (mL/min/1.73 m^2^)	75.7 ± 17.7	65.7 ± 20.6	56.7 ± 25.7	<0.001
TC (mg/dL)	164.5 ± 34.6	159.4 ± 32.9	168.8 ± 39.0	0.048
Triglycerides (mg/dL)	99.6 ± 66.1	119.6 ± 75.5	155.6 ± 107.4	< 0.001
Uric acid (mg/dL)	5.8 ± 1.6	6.2 ± 1.8	6.2 ± 2.0	0.147
C-reactive protein (mg/L)	1.1 ± 1.8	3.4 ± 5.7	4.1 ± 7.8	0.065
FGF21 (ng/L)	68.9 ± 24.0	163.8 ± 32.5	467.2 ± 304.8	<0.001
Cardiac catheterization				
Single vessel disease, *n* (%)	35 (19.8)	35 (19.8)	7 (4.0)	<0.001
Multiple vessel disease, *n* (%)	47 (26.6)	54 (30.5)	82 (46.3)	< 0.001
Mean blood pressure (mmHg)	104.7 ± 13.6	108.8 ± 16.6	104.7 ± 18.4	0.132
LVEF (%)	57.9 ± 8.8	56.0 ± 10.4	55.1 ± 11.9	0.047
Underwent PCI, *n* (%)	57 (32.2)	58 (32.8)	25 (14.1)	<0.001
Contrast volume (mL)	95.8 ± 73.1	93.3 ± 63.1	108.7 ± 79.5	0.117

BMI, body mass index; MI, myocardial infarction; ACEI, angiotensin-converting-enzyme inhibitor; ARB, angiotensin II receptor blocker; OHA, oral hypoglycemic agent; NAC, N-acetylcysteine; WBC, white blood cell; HbA1c, glycated hemoglobin; eGFR, estimated glomerular filtration rate; TC, total cholesterol; FGF21, fibroblast growth factor 21; PCI, percutaneous coronary intervention; LVEF, left ventricular ejection fraction.

### Study Endpoints and Survival

Thirty-four cases (6.4%) of CIN were identified at 48 hours after PCI, and 111 events (incidence, 13.6 [per 100-person-years]; 95% confidence interval [CI], 11.3–15.8) of renal function decline occurred during a mean follow-up period of 2.3 ± 1.3 years. Moreover, 88 cases (incidence, 7.66; 95% CI, 6.19–9.12) of major adverse cardiovascular even (MACE), including 58 cases of target vessel revascularization, 11 cases of non-fatal myocardial infarction (MI), and 19 cases of death, had occurred by the end of the study period. Patients in the higher FGF21 tertiles were found to have significantly higher percentages of CIN, and higher incidence of renal function decline as well as MACE (see Table 2). Log FGF21 was found to have slightly lower AUC than Mehran risk score (MRS), a published scoring system to predict the risk of CIN, but the difference did not achieve statistical significance (AUCs 0.753 vs. 0.770, *p* = 0.742). However, adding log FGF21 to MRS had showed better discriminatory performance than MRS alone in predicting the incidence of CIN (AUCs 0.782 vs. 0.770, *p* = 0.013). The above findings were summarized in the Supplement Fig. 1.

**Table 2:** Percentage of Contrast-Induced Nephropathy (CIN), and Incidence of Renal Function Decline, Major Adverse Cardiovascular Events (MACE) in Patients Grouped by Different Fibroblast Growth Factor 21 Concentration.

Outcome	T1	T2	T3	*P*
Cases underwent CAG	177	177	177	—
Cases of CIN	3	7	24	<0.001
Percentage of CIN (%)	1.7	4.0	13.6	<0.001
Person-years of follow-up	273.2	282.7	262.3	0.746
Cases of renal function decline	12	33	66	<0.001
Incidence of renal function decline (per 100-person-years, 95% CI)	4.4 (2.0–6.8)	11.7 (8.0–15.3)	25.2 (20.3–30.0)	<0.001
Cases of MACE	14	31	43	<0.001
Cases of revascularization	9	24	25	0.009
Cases of nonfatal MI	1	2	8	0.018
Cases of death	4	5	10	0.184
Incidence of MACE (per 100-person-year, 95% CI)	3.6 (1.8–5.4)	8.1 (5.5–10.7)	11.4 (8.4–14.3)	<0.001

T, tertile; CAG, coronary angiography;

95% CI, 95% confidence intervals.

**Figure 1:**
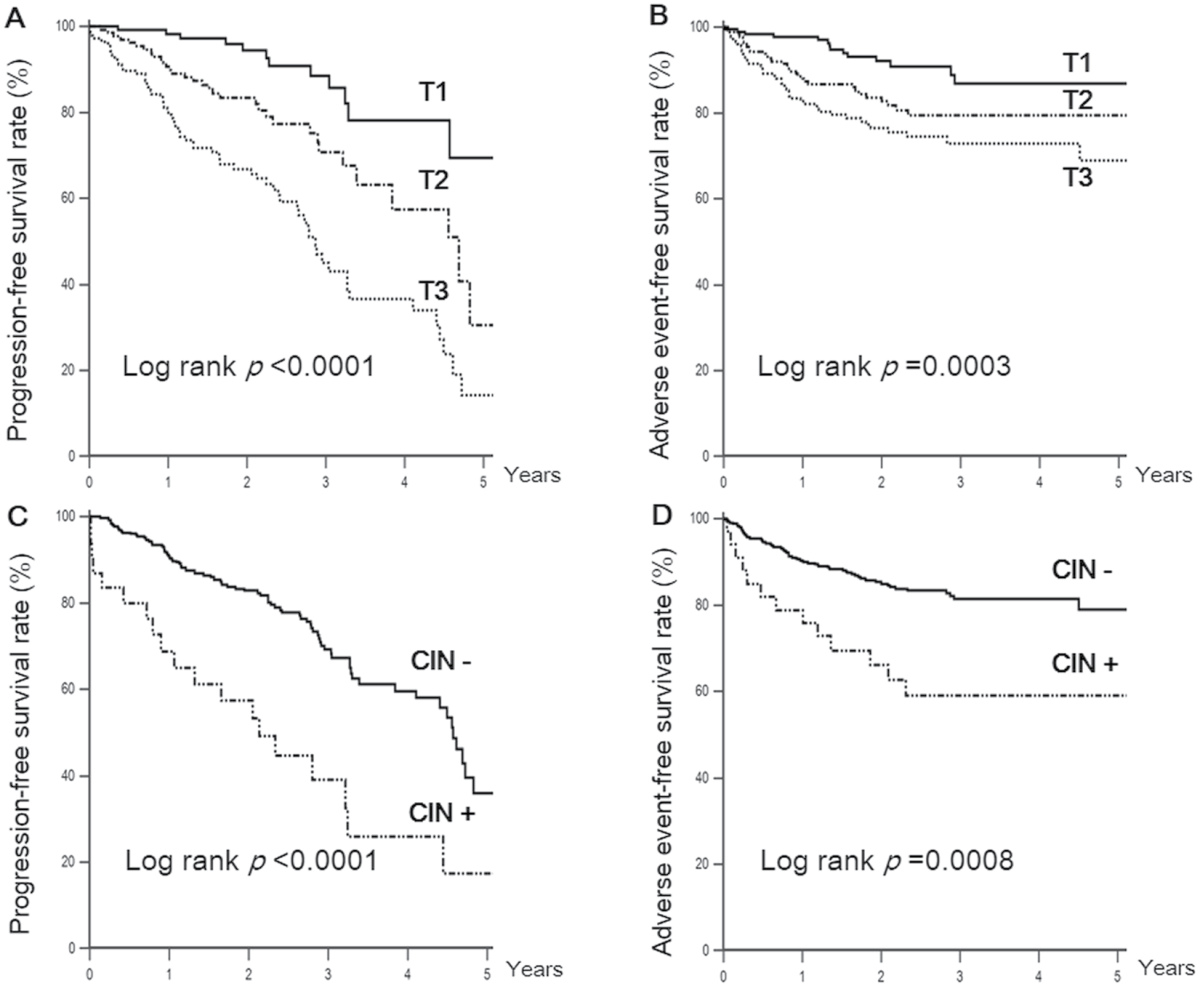
Kaplan–Meier curves of freedom from renal function decline and major adverse cardiac events by tertiles of serum fibroblast growth factor 21 concentration (**A**,**B**) and the incidence of contrast-induced nephropathy (**C**,**D**). T, tertile; CIN, contrast-induced nephropathy.

The Kaplan–Meier analysis demonstrated significantly lower survival from the adverse renal (log rank test, *p* < 0.0001) and cardiovascular events (log rank test, *p* = 0.0003) among subjects with higher FGF21 concentrations (Fig. 1A,B). In addition, subjects with documented CIN were found to have more occurrences of renal function decline (log rank test, *p* < 0.0001) and MACE (log rank test, *p* = 0.0008) after CAG (Fig. 1C,D).

In the multivariate regression analysis adjusted for age, gender, fasting blood sugar (FBS), baseline eGFR, hemoglobin, LVEF, contrast volume, and peri-procedure N-acetylcysteine (NAC) usage, serum FGF21 remained independently associated with the incidence of CIN (adjusted odds ratio, 4.66; 95% CI, 1.29–16.86; *p* = 0.019; Table 3). The Cox proportional-hazard analysis adjusted for age, gender, FBS, LVEF, eGFR, hemoglobin, proteinuria, and previous CIN revealed a strong positive association between the circulating FGF21 concentration and deterioration of renal function (adjusted hazard ratio [aHR], 7.98; 95% CI, 4.07–15.66; *p* < 0.001). The analysis of FGF21 level and MACE incidence yielded similar results. After adjustment for age, gender, FBS, LVEF, eGFR, hemoglobin, and undergoing PCI, FGF21 remained significantly associated with the occurrence of MACE. Circulating FGF21 concentration was an independent predictor of CIN, renal function decline, and MACE in patients with stable angina undergoing CAG and/or PCI.

**Table 3:** Multivariate Logistic Regression Analysis for FGF21 Value (Log Transformation before Analysis) to the Occurrence of CIN; and Cox Proportional Hazard Analysis for FGF21 Value to the Incidence of Renal Function Decline and MACE.

Variable	Univariate		Model 1*		Model 2^†^	
	HR (95% CI)	*P value*	HR (95% CI)	*P value*	HR (95% CI)	*P value*
**Contrast-induced nephropathy, n = 34**						
Log FGF21	15.58 (5.54–43.79)	<0.001	15.19 (5.36–43.09)	<0.001	4.66 (1.29–16.86)	0.019
Age	1.02 (0.99–1.05)	0.243	1.01 (0.98–1.04)	0.656	1.00 (0.96–1.03)	0.891
Male Gender	0.79 (0.39–1.61)	0.515	1.01 (0.48–2.12)	0.988	1.16 (0.50–2.69)	0.732
MBP	0.98 (0.95–1.01)	0.151				
FBS	1.00 (1.00–1.01)	0.058			1.00 (1.00–1.01)	0.030
LVEF	0.20 (0.01–5.53)	0.343				
eGFR	0.96 (0.94–0.97)	<0.001			0.99 (0.97–1.02)	0.030
Hemoglobin	0.58 (0.48–0.70)	<0.001			0.70 (0.55–0.89)	0.004
CRP	1.03 (0.98–1.09)	0.280				
Contrast volume	1.00 (1.00–1.01)	0.380				
NAC prevention	10.75 (4.76–24.27)	<0.001			1.63 (0.38–6.88)	0.509
Underwent PCI	1.18 (0.55–2.53)	0.677				
**Renal function decline, n = 111**						
Log FGF21	7.18 (4.35–11.88)	<0.001	7.00 (4.23–11.59)	<0.001	7.98 (4.07–15.66)	<0.001
Age	1.02 (1.00–1.03)	0.055	1.01 (1.00–1.03)	0.098	1.01 (0.99–1.03)	0.225
Male gender	0.74 (0.50–1.09)	0.124	0.97 (0.66–1.44)	0.888	1.07 (0.68–1.67)	0.777
MBP	1.00 (0.99–1.01)	0.911				
FBS	1.00 (1.00–1.00)	0.071			1.00 (1.00–1.00)	0.359
LVEF	0.03 (0.01–0.16)	<0.001			0.07 (0.01–0.42)	0.003
eGFR	0.98 (0.97–0.99)	<0.001			1.01 (0.99–1.02)	0.397
Hemoglobin	0.72 (0.65–0.79)	<0.001			0.79 (0.70–0.90)	<0.001
CRP	1.00 (0.97–1.04)	0.938				
Proteinuria	1.00 (1.00–1.01)	<0.001			1.00 (1.00–1.00)	0.913
Underwent PCI	0.76 (0.49–1.18)	0.757				
Previous CIN	2.90 (1.79–4.72)	<0.001			1.85 (1.03–3.30)	0.039
**Major adverse cardiovascular events, n = 88**						
Log FGF21	3.43 (1.97–5.95)	<0.001	3.40 (1.95–5.92)	<0.001	3.76 (1.80–7.86)	<0.001
Age	1.01 (0.99–1.03)	0.190	1.01 (0.99–1.02)	0.418	1.00 (0.98–1.02)	0.765
Male gender	0.98 (0.63–1.52)	0.917	1.12 (0.71–1.75)	0.628	1.05 (0.64–1.73)	0.857
MBP	1.00 (0.99–1.02)	0.548				
FBS	1.00 (1.00–1.01)	<0.001			1.00 (1.00–1.01)	0.010
LVEF	0.06 (0.01–0.33)	0.001			0.35 (0.05–2.74)	0.317
eGFR	0.98 (0.97–0.99)	<0.001			0.99 (0.98–1.00)	0.084
Hemoglobin	0.80 (0.72–0.90)	<0.001			0.96 (0.84–1.11)	0.590
CRP	1.01 (0.98–1.05)	0.444				
Underwent PCI	2.21 (1.45–3.38)	<0.001			2.15 (1.34–3.44)	0.002

*Adjusted age and gender.

^†^Adjusted age, gender, and variables with *p* values < 0.1 in univariate analysis.

FGF21, fibroblast growth factor 21; CIN, contrast-induced nephropathy; MACE, major adverse cardiovascular event; HR, hazard ratio; CI, confidence interval; MBP, mean blood pressure; FBS, fasting blood sugar; LVEF, left ventricular ejection fraction; eGFR, estimated glomerular filtration rate; CRP, C-reactive protein; NAC, N-acetylcysteine; PCI, percutaneous coronary intervention.

### Subgroup Analyses

**Figure 2:**
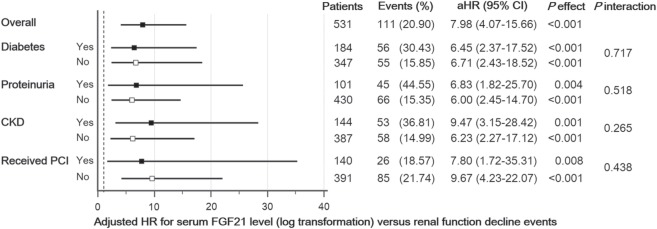
Subgroup analysis of the risk of renal function decline stratified by the presence of underlying diabetes, proteinuria, chronic kidney disease (eGFR < 60 mL/min/1.73 m^2^), and the status of percutaneous coronary intervention (PCI). eGFR, estimated glomerular filtration rate; aHR, adjusted hazard ratio; CKD, chronic kidney disease.

The study cohort was stratified by the presence of diabetes, proteinuria, CKD, and the status of PCI. As depicted in Fig. 2, increasing FGF21 concentration was significantly associated with renal function decline in all the different subgroups. Although HRs were higher for subjects with CKD, the difference were not significant (interaction *p* > 0.05). These results suggest that the association between circulating FGF21 concentration and renal function decline was independent of underlying disease, such as diabetes or CKD.

## Discussion

In this single-center, observational study involving 531 patients with stable angina undergoing elective CAG, circulating FGF21 concentration was associated with the incidence of CIN and subsequent renal function decline and adverse cardiovascular events during the mean follow-up period of 2.3 ± 1.3 years. Regardless of the presence of diabetes, the serum FGF21 concentration remained significantly associated with subsequent eGFR decline. To our knowledge, this longitudinal study is the first to explore the relationship between FGF21 and subsequent renal function deterioration in patients undergoing PCI. These results suggest that the circulating FGF21 level is an independent predictor of CIN and chronic renal function decline, and provide novel evidence of FGF21′s involvement in acute and chronic kidney function impairment.

CIN involves a series of complex interactions among different mechanisms. Contrast media may induce renal vasoconstriction via alterations in nitric oxide, endothelin, and adenosine, leading to subsequent renal medulla ischemia and acute tubular necrosis^9,10^. In addition, contrast media have cytotoxic effects via the upregulation of reactive oxygen species^9,11^ or direct induction of osmotic tubular nephrosis^4^. Although most cases of CIN are reversible, about 20% of those affected develop persistent renal damage^2^. In the Alberta registry^12^, CIN was associated with a 4-fold increased risk of progression to ESRD and a 2-fold increased risk of death after 39 months of follow-up. Our study produced similar results. Subjects with CIN tended to have increased incidence of comorbidities, which made them more vulnerable to adverse renal and cardiovascular events. Long-term renal function impairment may be a direct result of severe CIN or repeated kidney damage caused by the interaction among comorbidities, such as diabetes, hypertension, and CKD.

Accumulating evidence indicates that FGF21 is a novel polypeptide ligand that plays a critical role in glucose homeostasis and lipid metabolism^6^, and is a promising therapeutic target of diabetes^13^. Acting as an endocrine factor, FGF21 is secreted mainly by the liver via a peroxisome proliferator–activated receptor (PPAR)-α–mediated pathway under the stimulation of various nutrient stresses, such as starvation^14^, amino acid deprivation^15^, and consumption of a high-fat diet^16^. After binding to fibroblast growth factor receptor and β-Klotho, FGF21 promotes glucose uptake, fatty acid oxidation, and ketogenesis in adipocytes^7^. FGF21 was also found to be upregulated in numerous metabolic disorders, including diabetes^17^, obesity^16^, nonalcoholic fatty liver disease^18^, dyslipidemia, and coronary artery disease (CAD)^19^. Because of its regulatory property, an elevated circulating FGF21 level is usually considered to compensate metabolic dysfunction and tissue resistance. However, the precise role of upregulated FGF21 in metabolic disorders remains uncertain.

As a metabolic regulator in glucose and lipid homeostasis, FGF21 could be involved in diabetic kidney disease. Previous studies have shown that elevated FGF21 concentrations were associated with increased urine albumin exertion^20^ and long-term progress of eGFR decline in patients with type 2 diabetes^21^. In experimental and *in vitro* studies, Kim *et al*.^22^ reported that FGF21 and its receptor components were significantly upregulated in the mesangial cells of the kidneys in db/db mice, which suggests a theory of FGF21 resistance. In addition, administration of recombinant FGF21 decreased urinary albumin excretion and ameliorated morphologic glomerular abnormalities in db/db mice. Zhang *et al*.^8^ showed that exogenous FGF21 treatment prevented renal lipid accumulation, attenuated inflammation, and decreased oxidative stress in a mouse model of diabetic kidney disease. These findings support the renal protective effect of FGF21 in diabetic nephropathy.

In addition, an enhanced circulating FGF21 level has been shown to be associated with the deterioration of renal function^23,24^. This relationship was observed in community-dwelling adults^25^, independent of the presence of diabetes. In the Baltimore Longitudinal Study of Aging^25^, the correlation between FGF21 and renal function was consistent, even after the exclusion of all patients with diabetes (5.9% of 744 participants). The authors attributed this finding to the impaired renal elimination of FGF21 in subjects with CKD. Hindricks *et al*.^24^ further confirmed this hypothesis by observing a postsurgical surge in circulating FGF21 in patients who underwent unilateral nephrectomy. Another possible explanation is that elevated FGF21 is a compensation to impaired renal function in subjects with CKD, similar to the response to tissue resistance observed in diabetic nephropathy. Currently, no convincing evidence supports this hypothesis. Previous clinical studies were limited to clarification of the causal relationship because of their cross-sectional designs^23–25^. Our study demonstrated that the circulating FGF21 level was associated with the occurrence of CIN and subsequent renal function decline, regardless of the presence of diabetes. The association between FGF21 and renal function decline remained significant after adjustment for baseline eGFR level. These results suggest that elevation of the FGF21 concentration is more than a consequence of decreased renal elimination.

A possible explanation for the association between FGF21 concentration and non-diabetic kidney injury is based on the response to oxidative stress. FGF21 was recently suggested to be a regulator of mitochondrial and oxidative stress^26^. In the cell-line study, FGF21 exposure inhibited inflammation by attenuating the nuclear factor kB (NF-kB) signaling^27^. Metabolic disorders or chronic stress involving inflammatory responses would provoke FGF21 secretion as a compensatory response. In a rabbit model of critical illness, hepatic FGF21 expression was correlated with mitochondrial dysfunction and an integrated stress response marker^28^. Patients with critical illness were found to have increased circulating FGF21 levels, which were 8-fold higher than those of controls^28^. In the presented study, patients with elevated FGF21 concentrations may just have higher degrees of mitochondrial damage or oxidative stress before procedure, which made them more vulnerable to the CIN. Although oxidative stress is a co-factor of CIN and FGF21, we could not dissect the causal relationship based on indirect evidence. Whether oxidative stress can stimulate FGF21 elevation in subjects with CKD remains unknown, and more precise experimental studies are needed to reach a definitive conclusion on this issue.

This study had several limitations. First, it was a retrospective study, with a small number of cases and short follow-up period. Second, patients enrolled in our study were older and had a higher incidence of renal function decline compared with those participating in a previous study^21^. Caution should be taken while applying our findings to younger populations. Third, the renal endpoint was defined only by eGFR. Other clinical endpoints that interfere with renal outcomes, including newly diagnosed diabetes and the progression of proteinuria, were not included in the analysis. In addition, information about the longitudinal changes of FGF21 concentrations were absent since we did not routinely measured FGF21 during the follow-up period. Finally, confounding factors for CIN, such as peri-procedural hydration and exposure to nephrotoxic agents, could not be fully assessed due to the limited availability of data.

In conclusion, circulating FGF21 level was associated with the incidence of CIN and subsequent adverse cardiorenal events in patients who underwent CAG. These findings suggest that FGF21 may be an early predictor of CIN and subsequent renal function decline, and provide novel evidence of FGF21′s involvement in non-diabetic kidney disease.

## Methods

### Study Population

From December 2009 to March 2015, 588 subjects with stable CAD admitted for elective CAG and/or PCI to Taipei Veterans General Hospital were screened. In each case, the serum creatinine concentration was checked before CAG, and the eGFR was calculated using the CKD Epidemiology Collaboration (CKD-EPI) equation^29^. Patients with stage 5 CKD, defined as creatinine clearance <15 mL/min/1.73 m^2^, and those with pre-existing dialysis requirements were excluded from the analysis. Patients under the treatment of fenofibrate, which was known to be a PPARα agonist and may potentially affect circulating FGF21 concentrations^30^, were also excluded.

This research was conducted according to the principles expressed in the Declaration of Helsinki. It was approved by the research ethics committee of Taipei Veterans General Hospital, and all participants provided written informed consent.

### Baseline and Cardiac Catheterization Data Collection

The blood samples were acquired before CAG, and were centrifuged immediately to get serum. All patients were fasted for at least 8 hours before providing blood samples. The blood cell count and serum glucose, creatinine, uric acid, and lipid profiles were measured using routine laboratory methods. The urine protein concentration was assessed using commercial test strips. After measurement of clinical chemistries, the serum samples were stored in a −20 °C condition till the measurement of FGF21 in the batched assays about 1 week later. Serum concentrations of FGF21 were determined with a commercial enzyme-linked immunosorbent assay (R&D Systems, Inc., Minneapolis, MN, USA), as described previously^31^. The sensitivity was 7 ng/L. Intra- and interassay coefficients were 4.1% and 3.9%, respectively. Patients without detectable FGF21 levels were excluded from analysis.

After performing CAG, coronary angiograms were interpreted by two experienced interventional cardiologists. Coronary lesions with diameters showing >50% narrowing were considered to exhibit significant stenosis. The mean blood pressure (MBP) was measured with a pigtail catheter at the aortic root level. The left ventricular ejection fraction (LVEF) was estimated by left ventriculography. The contrast consumption of each patient was also recorded.

### Definition of Study Endpoints and Renal Function Decline

Serum creatinine was obtained for each patient before and 48 hours after procedure. For subjects with baseline creatinine more than 2.0 mg/dL, oral administration of *N*‐acetylcysteine (NAC, 600 mg twice daily) and intravenous hydration with 0.9% normal saline (1 ml/kg/hour) were given before and after CAG to prevent the occurrence of CIN. Nonionic low-osmolality contrast medium (iopromide) were used for all patients. The occurrence of CIN was defined as the post-procedural elevation of creatinine ≥0.5 mg/dL or ≥ 25% from baseline^21,32^. All subjects would be arranged to visit our outpatient department 1 week after the procedure, then every 3 months for a refill of medications. The follow-up process was performed and recorded by the same cardiologist. The observation ended on December 31^th^ 2016, or ended when the occurrence of a MACE, including target vessel revascularization, non-fatal MI, and death. Target vessel revasculization was defined as balloon dilatation or stent deployment over a previously treated lesion. Non-fatal myocardial infarction was defined as elevation of cardiac troponin I (>1 ng/ml) with ischemic symptoms. The detail definitions had been described in our previous work^33^.

**Figure 3:**
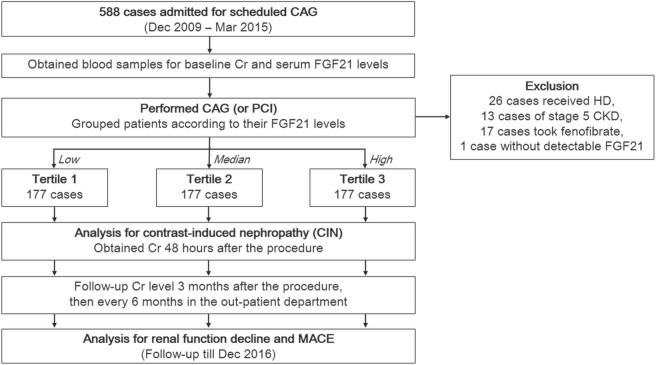
Flowchart of patient enrollment and follow-up. CAG, coronary angiography; PCI, percutaneous coronary intervention, HD, hemodialysis; CKD, chronic kidney disease; FGF21, fibroblast growth factor 21, MACE, major adverse cardiovascular events.

Clinical chemistry data, including the creatinine value, were obtained 3 months after the procedure, then every 6 months during the follow-up period. Renal function decline was defined as more than 30% reduction of eGFR from baseline^34^. After renal function declined was identified, repeated measurement of creatinine would be performed 1 month later to ensure accuracy of diagnosis. The flowchart of patient enrollment and follow-up was depicted in Fig. 3. Each patient’s chart was reviewed in detail to collect the medical data.

### Statistical Analysis

The enrolled subjects were grouped into tertiles according to serum FGF21 concentration. Clinical and laboratory data were compared using analysis of variance for continuous variables (expressed as means ± standard deviations) and the chi-squared test for categorical variables (expressed as counts and percentages). Occurrence CIN was surveyed and calculated for all enrolled subjects. Areas under the ROC curves (AUCs) were used to evaluate of the predictive accuracy of FGF21 and MRS^35^ in prediction the incidence of CIN. Pairwise comparison between 2 AUCs was performed with the method of DeLong *et al*.^36^. Survival curves were generated using the Kaplan–Meier method, and survival was compared among groups using the log-rank test. Univariate analysis was performed for FGF21 and variables that known to be risk factors of CIN^35^, renal function decline, or MACE^37^, including age, gender, mean blood pressure, FBS, LVEF, eGFR, hemoglobin, C-reactive protein, NAC prevention, the status of PCI, contrast volume (for CIN), proteinuria, and the occurrence of CIN (for eGFR decline). Variables with statistical significance in the univariate analysis were further entered into the multivariate models. Multivariate logistic regression analysis (for CIN) and Cox proportional-hazard regression analysis (for eGFR decline and MACE) were conducted to assess the independent effects of FGF21 concentration. To investigate the effect of FGF21 on renal function decline modified by different comorbid conditions, we performed subgroup analyses with stratification according to the presence of diabetes, proteinuria, CKD (defined as eGFR < 60 mL/min/1.73 m^2^), and the status of PCI. Interactions between FGF21 concentration and comorbid conditions were examined by adding a product term to the Cox regression analysis. Data were analyzed using SPSS version 18.0 (SPSS Inc., Chicago, IL, USA) and MedCalc version 11.4.2.0 (MedCalc Software, Mariakerke, Belgium). *P* values < 0.05 were considered to be statistically significant.

### Data Availability

The datasets generated during and/or analysed during the current study are available from the corresponding author on reasonable request.

## Electronic supplementary material

Supplement Figure 1
