# Author Correction: Circulating Fibroblast Growth Factor 21 is Associated with Subsequent Renal Injury Events in Patients Undergoing Coronary Angiography

**DOI:** 10.1038/s41598-018-35360-0

**Published:** 2018-11-15

**Authors:** Cheng-Hsueh Wu, Ruey-Hsing Chou, Chin-Sung Kuo, Po-Hsun Huang, Chun-Chin Chang, Hsin-Bang Leu, Chin-Chou Huang, Jaw-Wen Chen, Shing-Jong Lin

**Affiliations:** 1 Division of Cardiology, Department of Medicine, Taipei Veterans General Hospital, Taipei, Taiwan; 2 Division of Endocrinology and Metabolism, Department of Medicine, Taipei Veterans General Hospital, Taipei, Taiwan; 3 Department of Critical Care Medicine, Taipei Veterans General Hospital, Taipei, Taiwan; 4 Healthcare and Management Center, Taipei Veterans General Hospital, Taipei, Taiwan; 5 Department of Medical Education, Taipei Veterans General Hospital, Taipei, Taiwan; 6 Department of Medical Research, Taipei Veterans General Hospital, Taipei, Taiwan; 7 Cardiovascular Research Center, Taipei Veterans General Hospital, Taipei, Taiwan; 8 Institute of Clinical Medicine, National Yang-Ming University, Taipei, Taiwan; 9 Institute of Pharmacology, National Yang-Ming University, Taipei, Taiwan; 10 School of Medicine, National Yang-Ming University, Taipei, Taiwan

Correction to: *Scientific Reports* 10.1038/s41598-018-30744-8, published online 20 August 2018

The original version of this Article omitted an affiliation for Chen-Huseh Wu. The correct affiliations for Chen-Huseh Wu are listed below:

Division of Cardiology, Department of Medicine, Taipei Veterans General Hospital, Taipei, Taiwan

Department of Critical Care Medicine, Taipei Veterans General Hospital, Taipei, Taiwan

School of Medicine, National Yang-Ming University, Taipei, Taiwan

This has now been corrected in the HTML and PDF versions of this Article, and in the accompanying Supplemental Material.

