# Supplementary material for: Circulating Fibroblast Growth Factor 21 is Associated with Subsequent Renal Injury Events in Patients Undergoing Coronary Angiography
Source: Sci Rep. 2018 Aug 20;8:12425. doi: 10.1038/s41598-018-30744-8 (PMC6102249; doi:10.1038/s41598-018-30744-8)
Supplement: Supplementary file 1 — Supplement Figure 1 [file 41598_2018_30744_MOESM1_ESM.pdf]

SUPPLEMENT

Circulating Fibroblast Growth Factor 21 is Associated with Subsequent Renal Injury Events in Patients Undergoing Coronary Angiography

Cheng-Hsueh Wu[1,3,10], Ruey-Hsing Chou[1,3,7,8], Chin-Sung Kuo[2,7,8,*], Po-Hsun Huang[1,3,7,8,*], Chun-Chin Chang[1,7,8], Hsin-Bang Leu[1,4,7,8], Chin-Chou Huang[1,5,7,9], Jaw-Wen Chen[1,6,7,9], Shing-Jong Lin[1,4,7,9]

[1] Division of Cardiology, Department of Medicine, Taipei Veterans General Hospital, Taipei, Taiwan;

[2] Division of Endocrinology and Metabolism, Department of Medicine, Taipei Veterans General Hospital, Taipei, Taiwan;

[3] Department of Critical Care Medicine, Taipei Veterans General Hospital, Taipei, Taiwan;

[4] Healthcare and Management Center, Taipei Veterans General Hospital, Taipei, Taiwan;

[5] Department of Medical Education, Taipei Veterans General Hospital, Taipei, Taiwan;

[6] Department of Medical Research, Taipei Veterans General Hospital, Taipei, Taiwan;

[7] Cardiovascular Research Center, Taipei Veterans General Hospital, Taipei, Taiwan;

[8] Institute of Clinical Medicine, National Yang-Ming University, Taipei, Taiwan;

[9] Institute of Pharmacology, National Yang-Ming University, Taipei, Taiwan.

[10] School of Medicine, National Yang-Ming University, Taipei, Taiwan.

Supplement Figure 1. The C-statistics of Mehran risk score (MRS) and fibroblast growth factor 21 (FGF21) in prediction the occurrence of contrast-induced nephropathy in patients underwent coronary angiography. The area under the ROC curve (AUC) was used as a measure of the predictive accuracy of MRS and FGF21. The statistical significance of pairwise comparison between 2 AUCs was tested with the method of DeLong *et al*.[1]

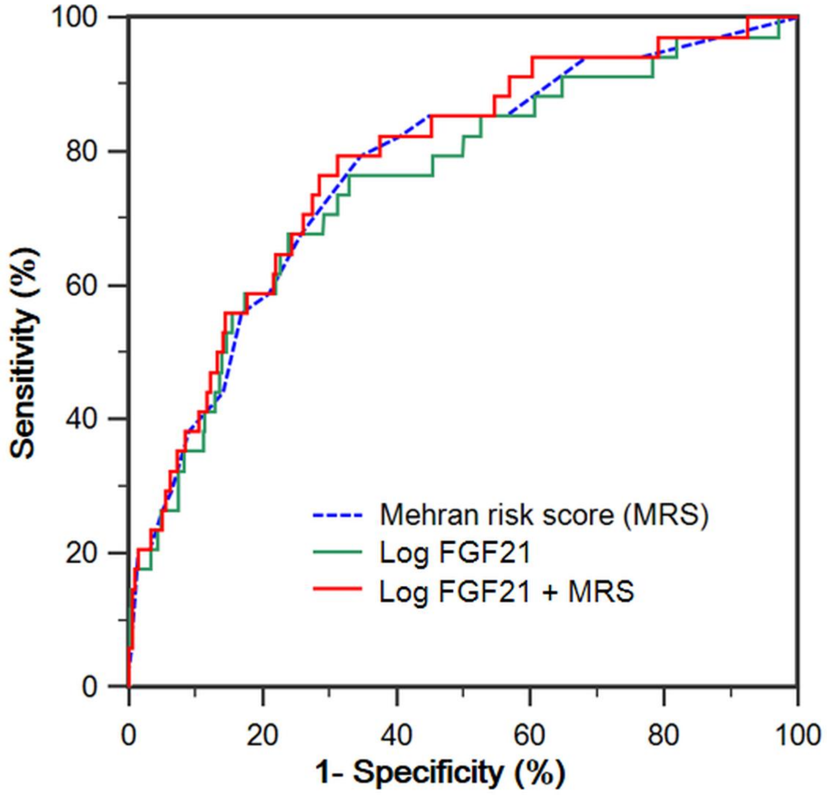

	AUCs (95% CI)	
Meharn risk score (MRS)	0.770 (0.732-0.806)	
Log FGF21	0.753 (0.714-0.789)	
Log FGF21 + MRS	0.782 (0.744-0.816)	
	**Difference between AUCs**	***P* value**
Log FGF21 vs. MRS	-0.018 (-0.089 to 0.124)	0.742
Log FGF21 + MRS vs. MRS	0.029 (0.002 to 0.020)	0.013

Reference:

1. DeLong, E.R., *et al*. Comparing the areas under two or more correlated receiver operating characteristic curves: A nonparametric approach. *Biometrics*. **44**, 837-845 (1988)
